# Research on named entity recognition method of marine natural products based on attention mechanism

**DOI:** 10.3389/fchem.2023.958002

**Published:** 2023-02-08

**Authors:** Xiaodong Ma, Rilei Yu, Chunxiao Gao, Zhiqiang Wei, Yimin Xia, Xiaowei Wang, Hao Liu

**Affiliations:** ^1^ College of Computer Science and Technology, Ocean University of China, Qingdao, China; ^2^ Pilot National Laboratory for Marine Science and Technology, Qingdao, China

**Keywords:** named entity recognition, marine natural products, attention mechanism, inflated convolutional neural network, conditional random field

## Abstract

Marine natural product (MNP) entity property information is the basis of marine drug development, and this entity property information can be obtained from the original literature. However, the traditional methods require several manual annotations, the accuracy of the model is low and slow, and the problem of inconsistent lexical contexts cannot be solved well. In order to solve the aforementioned problems, this study proposes a named entity recognition method based on the attention mechanism, inflated convolutional neural network (IDCNN), and conditional random field (CRF), combining the attention mechanism that can use the lexicality of words to make attention-weighted mentions of the extracted features, the ability of the inflated convolutional neural network to parallelize operations and long- and short-term memory, and the excellent learning ability. A named entity recognition algorithm model is developed for the automatic recognition of entity information in the MNP domain literature. Experiments demonstrate that the proposed model can properly identify entity information from the unstructured chapter-level literature and outperform the control model in several metrics. In addition, we construct an unstructured text dataset related to MNPs from an open-source dataset, which can be used for the research and development of resource scarcity scenarios.

## 1 Introduction

The special high-salt, high-pressure, and low-temperature living environment of the ocean has led to the formation of many marine natural products (MNPs) with novel structures and unique effects, and the drugs developed using these MNPs have unique effects on antitumor, antioxidant, and immunity enhancement (Lu et al., 2021). So far, more than 38,000 MNPs with novel structures and diverse activities have been discovered (Li et al., 2022a). However, in the early stage of drug research and development based on MNPs, it is extremely dependent on obtaining relevant research data from various data sources and numerous documents. Its application plays a key role in deepening the connection between entities and attributes. Therefore, the optimization of named entity recognition methods is the top priority of research in the early stages of marine drug development (Ghareeb et al., 2020).

The number of MNP literature is growing rapidly; for example, PubMed (Ma et al., 2022) contains more than 13,000 articles with the keyword “Marine Natural Products,” and MarinLit (Zhang et al., 2022) contains more than 38,000 articles. However, few high-quality datasets specialize in MNP research, and there are problems such as limited data types and numbers and a lack of annotations (Lample et al., 2016). Therefore, it is particularly important to automatically obtain the attribute information on entities from the original literature and label them by themselves (Bonner et al., 2021). The application of Named Entity Recognition (NER) technology greatly improves the extraction efficiency of key information in the field of MNPs. Named entity recognition is designed to automate the identification of entity information in a domain-specific text, which can further reduce the workload of researchers in data processing and application (Cong et al., 2018). However, named entity recognition only categorizes the recognized entity and attribute information and cannot closely link entities to entities and entities to attributes. The current named entity recognition methods need to be strengthened for consistent recognition of contextually annotated entities.

Knowledge graph technology is very important for marine drug development and drug-assisted design, and the use of knowledge graph technology can greatly improve the efficiency of new drug development and reduce the cost of drug development (Sang et al., 2018). According to the survey, data in the field of marine natural products are characterized by multiple sources, heterogeneity, ambiguity, and other aspects. As a large network connecting various semantic relationships between entities and concepts, knowledge graphs can help unify and standardize entities encoded by different identifiers and fuse data from multiple heterogeneous sources. In addition, as knowledge graphs emphasize the coverage of entities, they can keep the number of points and edges huge to reach a relatively large scale in the face of the huge scale of MNP domain data. However, the current MNP knowledge graph construction mainly relies on the annotated entities or relationships and rarely involves the automated direct acquisition of entities, attributes, and attribute values from unstructured text, which is difficult to meet the requirements of building large-scale knowledge graphs (Shao et al., 2021).

Moreover, there are mainly three difficulties in the field of MNPs using named entity recognition technology to extract entities in the literature:

(1) In terms of the construction of MNP datasets, MNP data information has a wide range of sources and various data types. It is difficult to obtain relevant field information data from multiple data sources and integrate them.
(2) In terms of the textual features of MNPs, there are inconsistencies in the abbreviations, proper nouns, conjunctions, and full-text annotations in the related literature, resulting in a low recognition effect.
(3) From the perspective of the recognition accuracy of named entities in the MNP literature, the current mainstream named entity recognition methods in the field of biomedicine are inconsistent in the recognition of context-annotated entities in several MNP literature studies.

**FIGURE 1:**
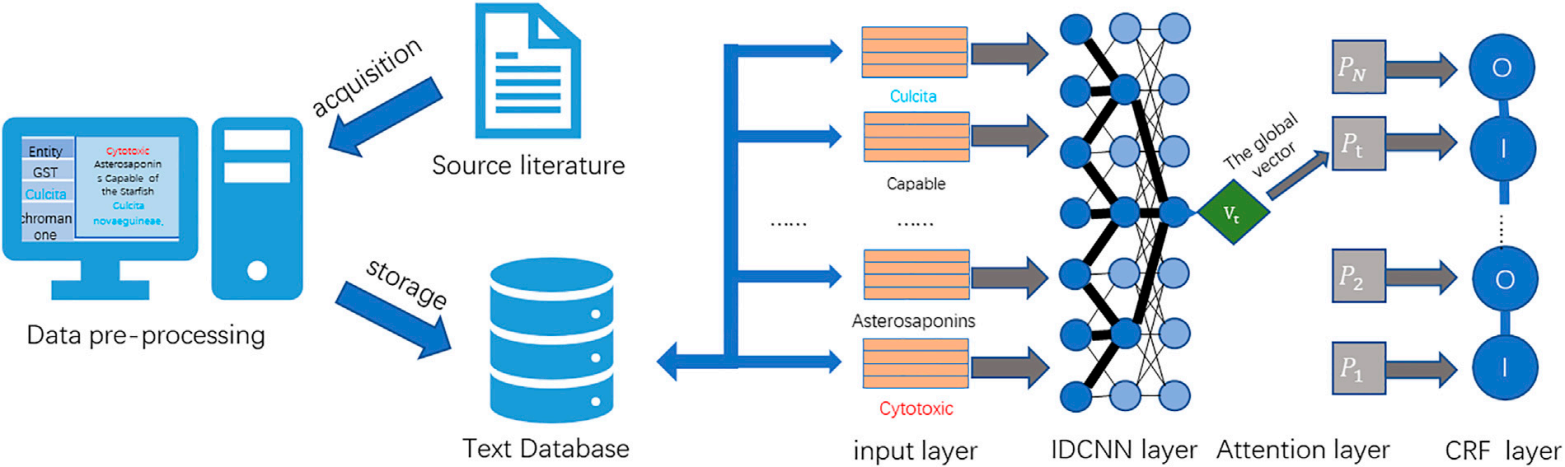
Overall technology roadmap.

In order to solve the aforementioned problems, this study proposes an attention mechanism-based named entity recognition technology to serve the field of MNPs. First, the need for named entity recognition in the extensive literature in the field of MNPs is studied. A high-quality dataset of MNPs is constructed combined with data from MNPs and related biomedical fields. Then, various deep learning-based named entity recognition methods (Luo et al., 2017; Yadav et al., 2019; Li et al., 2021) are studied, and an ATT-IDCNN-CRF method is improved and proposed. The method has good performance in terms of training efficiency and recognition effect on the dataset proposed in this study and basically solves the difficulty of named entity recognition in the field of MNPs. The overall technology roadmap for this study is shown in Figure 1.

## 2 Related work

MNP databases are an important reference for marine drug discovery and development, and the number of databases is gradually increasing. MarinLit and the Marine Natural Products Dictionary are currently the most exhaustive and complete MNP databases. However, the need for subscriptions and the limitation to payment reduce the breadth of academic research. Marin Chem 3D (July 2018) is the world’s first three-dimensional structure database of MNPs. Although there are not much activity data, it contains more than 30,000 three-dimensional structures of MNPs. CMNPD (Lyu et al., 2021) is also a very comprehensive database of more than 31,000 chemical entities with a wide range of physical, chemical, and pharmacokinetic properties; standardized bioactivity data; systematic classification; and geographical distribution of source organisms and detailed literature citations, which were constructed from the initial manual acquisition of information on entity properties in the literature by experts to the later application of named entity identification methods to automate the identification of desired entities from the original literature. Although the most widely used convolution neural network (CNN) (Aslan et al., 2021) has obvious computational advantages in the named entity recognition of MNP documents, the traditional CNN can only obtain a small part of the input text information after convolution. In order to obtain contextual information, more convolutional layers need to be added, resulting in deeper networks, more parameters, and being prone to overfitting (Fang et al., 2020). Inflated convolutional neural network (IDCNN) (Li et al., 2022b) adds convolution holes to CNN, which enables IDCNN to control its sliding window to omit inputs of a specific length range, which can reduce the number of convolutional layers to better capture sentence context information and greatly improve the efficiency of parallel computing (Wang and Xu, 2017). However, its recognition accuracy for long-named entities is not high. Long short-term memory networks (LSTMs) (Ma et al., 2021) are a special type of recurrent neural networks (RNNs) that can learn long-range dependencies. Currently, the BiLSTM-CRF model based on bidirectional LSTM (BiLSTM) combined with CRF has become the most mainstream model in deep learning-based NER methods (Wu et al., 2019). In terms of features, the model inherits the advantages of deep learning methods. It can achieve good results using word and character vectors without feature engineering. If there are high-quality dictionary features, it can be further improved (Wang et al., 2018). The accuracy of the named entity recognition model will directly determine the success of knowledge graph construction and entity activity relationship prediction (Zeng et al., 2017). Knowledge graph construction is also a key step in database establishment. The construction of early knowledge graphs was manually constructed by experts in related fields, such as WordNet (Miller, 1995), Cyc (Lenat and Douglas, 1995), and OpenCyc (Färber et al., 2018), but it is extremely labor-intensive. With the establishment of the World Wide Web, it has entered the era of a semantic network, in which DBpedia (Auer et al., 2007) and Yago (Rebele et al., 2016) are the representatives that combine entities and relationships into a semantic network. However, it still needs to be built manually. The era of knowledge graphs has emerged to effectively search and analyze knowledge and apply entities in other aspects. Typical uses of knowledge graphs for MNPs include CMNPD (Lyu et al., 2021), MC3D, and Marine Chinese Medicine Knowledge Graph (Liu and Li, 2021). However, the biological attribute information in the MNP knowledge graph system remains lacking and needs to be supplemented.

This study aims to solve the construction of datasets and the optimization of named entity recognition models during the construction of the MNP knowledge graph system. We constructed an unstructured text dataset related to MNPs from open-source datasets for research and development in resource-scarce scenarios. In terms of method, a named entity recognition method based on the attention mechanism, inflated convolutional neural network (IDCNN), and CRF is proposed, which can automatically identify entity attribute information in the MNP literature using attention. The mechanism to obtain full-text-level context information improves the situation of inconsistent recognition results of the same word. Experiments demonstrate that the proposed model can properly identify entity information from the unstructured chapter-level literature and outperform the control model on multiple metrics.

## 3 Construction of a marine natural product dataset

Few datasets specialize in MNP domain research, and the related data sources have high charges, imperfect data, and low retrieval efficiency. A diverse, data-rich, and strong correlation of the MNP domain dataset is created in this study to verify the effectiveness of the proposed method.

### 3.1 Dataset acquisition

To provide entity-relationship dependence of datasets in MNPs and related biomedical fields, we selected more than 30 existing public databases in the field of marine biomedicine for our study and finally selected the literature searched in PubMed with “Marine natural product” as the keyword. The abstracts in PubMed and some abstracts in MarinLit were selected. In addition, this study also includes some annotated data provided by the laboratory team. Therefore, in addition to investigating these databases in the field of MNPs and related pharmaceuticals, we need to use specific methods to obtain data from different data sources and organize and integrate these data to build a literature dataset in the field of MNPs.

### 3.2 Data preprocessing

Due to the diverse structure of the acquired data, it cannot be directly used for dataset construction and named entity recognition experiments. Converting these data to the same form is an important basis for improving the accuracy of subsequent work (Davis et al., 2011). Moreover, the experimental process of named entity recognition in this study is relatively complex, and the processing time is long. Therefore, this study preprocesses the literature used for entity extraction. In order to reduce the interference factors to the experiment and improve the accuracy and operation efficiency of the experimental results, this study excludes irrelevant sentences from the data and only retains sentences with valid entity results in the data. The specific process is as follows: first, this study splits the sentences of the literature, then performs simple formatting processing on them (e.g., adding breaks and punctuation and removing extra spaces), and omits sentences that do not have valid entities and are inappropriate in length. After that, a data pair of <entity, type > is created for entity information, and an index is built on the data pair. Finally, the indexed data pairs are retrieved and paired with the preprocessed sentences.

### 3.3 Data storage

**FIGURE 2:**
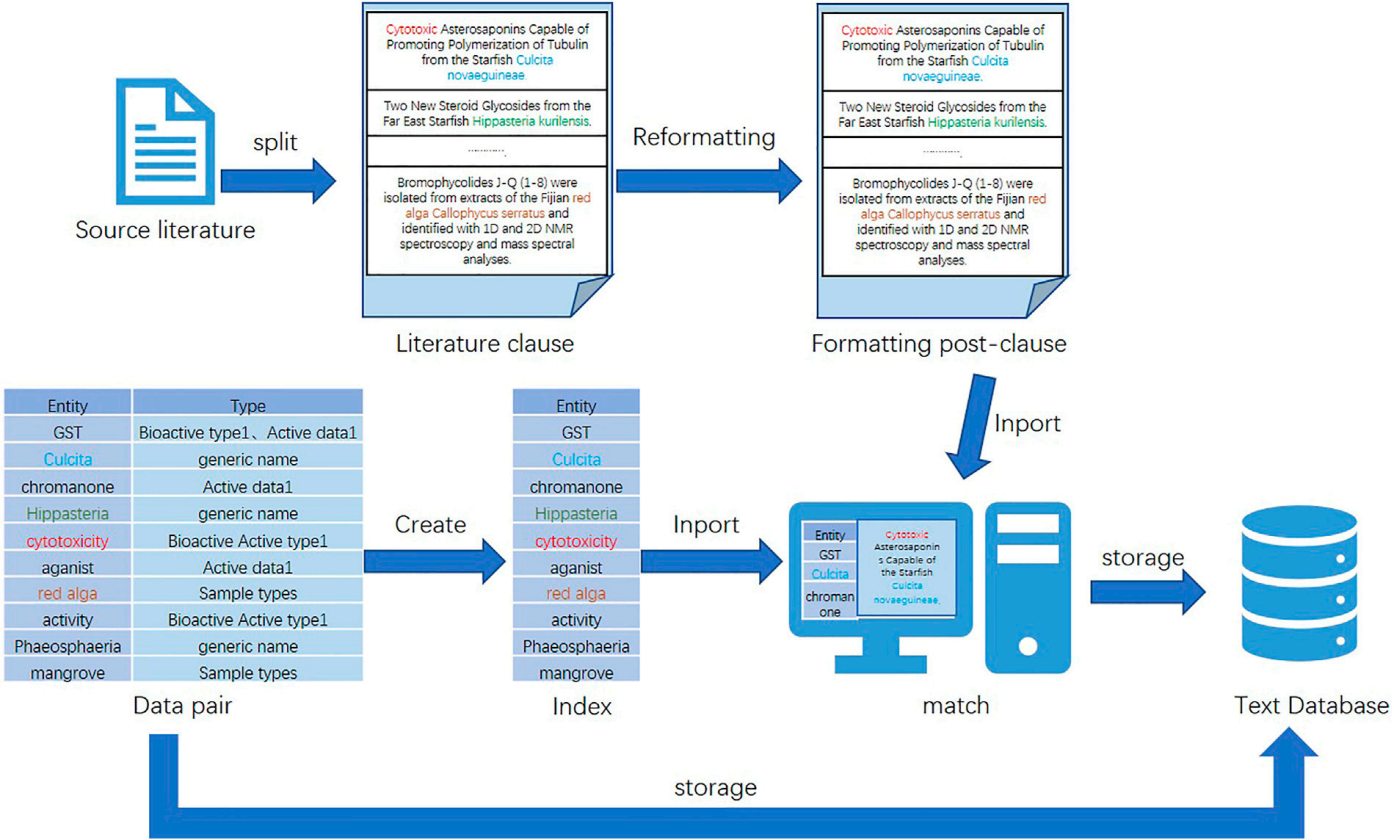
Dataset preparation process.

After the data preprocessing is completed, the data should be stored. Generally, the dataset can be stored in a text or data table form. The database of the data table type includes a relational database. Text databases can be used to store data in any text form. The latter are mainly relational databases, type tables, and triple tables. The storage of these three types of table structures has the advantage of being easy to understand and operate. However, once the data scale becomes larger or the operation becomes more complex, the advantages of such structures will disappear. Instead, it will be characterized by high overhead, low practicability, and low efficiency. The advantage of using a text database is as follows: when faced with large-scale data, it can properly combine text algorithms to improve the efficiency of complex queries and strong information description capabilities. Therefore, in the face of the large volume of data in this study, out of consideration of performance, stability, compatibility, and other characteristics, this study chooses the text database as the storage database of the dataset. A diagram of the dataset preparation process for this study is shown in Figure 2.

## 4 Improved named entity recognition method

### 4.1 IDCNN-CRF architecture

According to the actual needs, this study selects the current high-efficiency dilated convolution model (IDCNN-CRF). IDCNN increases the field of view of the convolution exponentially, but the parameters used increase linearly, which can fully utilize the power of GPU parallel computing, so compared with other models, the use of IDCNN-CRF can greatly speed up the training speed.

Similar to most traditional machine learning NER methods, the IDCNN-CRF method is a sentence-level NER method (Sagar et al., 2013). A common problem at the sentence level is that the contextually labeled entities may be inconsistent. In order to address this issue, previous work often employs a rule-based post-processing approach to enforce label consistency (Kim et al., 2019). However, if the label is misidentified in the front, it will greatly increase the misjudgment of subsequent labels (Mendez et al., 2019). In order to avoid the errors caused by the aforementioned methods, this study introduces the attention mechanism on the basis of the original IDCNN-CRF model. The attention mechanism can use the part-of-speech of words to weigh the extracted features to improve the consistency of contextual annotation entities.

### 4.2 Introduction of the attention mechanism

As the current point in the MNP literature has a low correlation with some long-distance information, even if the receptive field area of the dilated convolution is increased to obtain long-distance information, the data cannot have a high degree of accuracy consistency. In order to improve the accuracy, the named entity recognition task in this study needs to obtain full-text-level content information as much as possible. Therefore, this study uses the attention mechanism to improve the IDCNN-CRF model. First, in order to achieve the purpose of weight summation, the model will change the word vector and word vector in the way of combination. Then, in order to achieve the purpose of learning the attention weight, a hidden layer with two layers is used for combined learning. In this way, the model can use both word vector and character vector information to non-statically obtain text-level unstructured context-dependent relevant information (Luo et al., 2018).

In the process of entity extraction, the sentence in the document is first converted into a sequence of word vectors, and the sequence of word vectors is used to generate character vectors, and then, a matrix of character vectors is constructed. To obtain character-level features for each word, IDCNN applies convolution and pooling to a matrix of character vectors. By combining the word vector and character vector of each word, the input sequence of the IDCNN layer is formed, which is finally input into the network.

After analysis, the input file $D=\left(X_{1},X_{2},\ldots ,X_{m}\right)$ consists of *m* sentences; each sentence is represented as $X=\left(X_{1},X_{2},\ldots ,X_{n}\right)$ , where *n* is the number of words in the sentence. Like the regular IDCNN-CRF model, the embedding vector transformed using the word2vec tool is first provided as the input to the IDCNN layer. Then, in addition to the original IDCNN layer, an additional layer is added—the attention layer. The newly added layer acts as an attention mechanism in the constructed model, using the Attention layer in the document to explore the similarity between tags. To this end, the attention similarity matrix S is specially introduced here, and the attention similarity matrix S is used to calculate the similarity between the tags we are concerned about and each tag in the document. The similarity is expressed by the attention degree. Among them, in the attention matrix S, we can obtain the weight required by the attention matrix S by comparing the representation of the attention word *i* and the *j*th word in the document as follows:

$$s_{i,j}=\frac{\exp\left(f\left(x_{i},x_{j}\right)\right)}{\sum_{m}^{i}\exp\left(f\left(x_{i},x_{m}\right)\right)}.$$ (4–1)

$f\left(x_{i},x_{j}\right)$ is the function of the outputting probability value, which acts as an alignment function. To facilitate data processing and speed up computation, we use tanh⁡⁡ to compute the value of $f\left(x_{i},x_{j}\right)$ :

$$f\left(x_{i},x_{j}\right)=\tanh\left(\left.M_{s}{\left[x\right.}_{i};x_{j}\right]\right).$$ (4–2)

In the aforementioned score function calculation formula, $M_{s}$ is called the weight transformation matrix, equivalent to a parameter in the model. Then, the output value $q_{j}$ of each IDCNN is weighted and summed, and the weighted sum is assigned to the vector $v_{i}$ acting on the whole document:

$$v_{i}=\sum_{j=1}^{N}s_{i,j}q_{j}.$$ (4–3)

The following step is to generate the output of the attention layer. The specific steps are as follows: first, concatenate the global vector $v_{i}$ and the output value $q_{j}$ of IDCNN, then feed the concatenated value to the tanh⁡⁡ function, and finally generate the attention layer output:

$$p_{i}=\tanh\left(M_{v}\left[v_{i};q_{j}\right]\right).$$ (4–4)

Then, predict the activation function score for the word. This score is predicted by the tanh function value obtained in the previous step:

$$e_{i}=\tanh\left(S;p_{i}\right).$$ (4–5)

In this model, we first perform two regular 1D convolution operations on the output of the word embedding layer and then perform a dilated convolution operation to replace the max pooling layer in a standard convolutional neural network, and finally put the attention mechanism layer. The tanh layers are output as two fully connected layers.

At the end of the MNP entity extraction work, we use the CRF layer in the IDCNN-CRF model to select the best path, as shown in Eqs. 4–6, where *R* is the label information transfer matrix of this model and *O* is used as this model. The data score result matrix of the final calculated score result will be calculated by the comprehensive calculation of the input document *D* and *y* in all label paths, and an optimal label transfer path is decoded:

$$L\left(D,y\right)=\sum_{m}\sum_{i=1}^{n}\left(R_{y_{i}-1,y_{i}}+O_{i,y_{i}}\right).$$ (4–6)

### 4.3 ATT-IDCNN-CRF model

**FIGURE 3:**
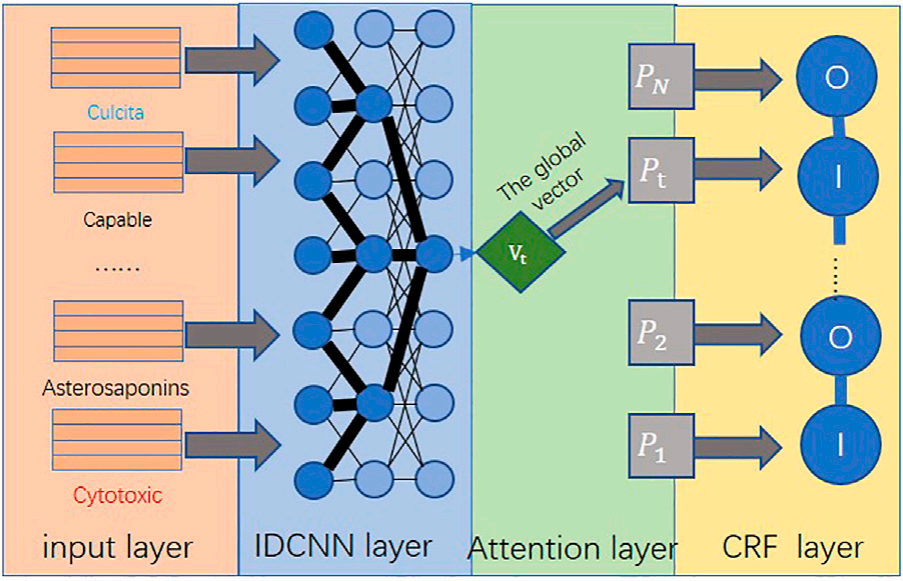
Improved ATT-IDCNN-CRF model architecture.

The ATT-IDCNN-CRF model constructed in this study can fully guarantee the contextual information on the text at the same time and make the model parameters not too much to cause overfitting. Therefore, the model can improve the training speed while ensuring the accuracy of text feature extraction. ATT-IDCNN-CRF is mainly composed of the attention, IDCNN, and CRF layers. The architecture of the model in this study is shown in Figure 3.

## 5 Result

### 5.1 Dataset

The training sets of the experiments in this study are mainly the MNP text dataset and the corpus CHEMDNER of BioCreative IV in the biological field (Chollet, 2017). The test set is the MNP text dataset proposed in this study. Among them, the data entity types comprise dozens of types, including unrelated samples, such as compounds, targets, small drug molecules, diseases, proteins, documents, and drugs, as well as the attributes of the aforementioned entities (Karmakar et al., 2021). The literature is divided into 37,926 training datasets and 7,586 testing datasets. Table 1 shows information about complete entity types and examples.

**TABLE 1:** Complete entity types and some examples.

Entity type	Sample
Formula	C_60_H_94_O_34_S_2_Na_2_ and C_35_H_42_O_11_
Sample source	The coast of Key Largo, off Ulleung Island, Korea
Species	*Theonella* and *moluccensis*
Generic name	*Siliquariaspongia* sp. and *Xylocarpus*
Active data	MIC80, <50.0
Periodical information	Molecules 2009, 14, 414–422, J. Nat. Prod. 2009, 72, 1657–1662
Compound name	Lepirudin and S-sulfocysteine
Relative molecular mass	201.221 and 156.850
Name-in-notebook	1987–2, 1987–3
Smiles	c1cc (c2c (c1)C (=O)c1c (C2 = O)cc2c (c1O)cc (cc2O)C)O
Molecular weight	684.4210 and 320.3040
Name	Prosurugatoxin and surugatoxin
Number of heavy atoms	9, 24
Number of rotatable bonds	3
donorHB	9, 1
PSA	112.0280
AlogP	−2.9260 and 3.4010
accptHB	18, 5
Species and origin	Digestive gland of *B. japonica*
Action	Prosurugatoxin evoked mydriasis in mice at a minimum effective intraperitoneal dose of 15 ng/g body weight and inhibited the contractile response of isolated guinea pig ileum induced by 3 × 10-5 g/ml of nicotine at a concentration of 5 × 10-9 g/ml
PercentHumanOralAbsorption	0.0000 and 77.0340
PMDCK (nm/s)	0.4750 and 47.3340
Pcaco (nm/s)	0.6670 and 114.0480
logS (S in mol/L)	−3.1160 and −4.1410
logHERG (IC_50_)	−4.1280 and −5.0440
logBB	−3.7450 and −1.4770
logKp	−8.5020 and −4.1240
logKhsa	−0.8550 and 0.2310

### 5.2 Evaluation criteria

Equations (5–1), (5–2), and (5–3) give the calculation method of the model evaluation index and the meaning of the corresponding parameters: $T_{p}$ represents the real sample, $F_{p}$ represents the false positive sample, $T_{n}$ represents the true negative sample, and $F_{n}$ represents a false negative sample. We define the samples whose entity types and locations in the test samples are the same as the model output judgment results as real samples; the samples whose output result is that the entity can be recognized, but the category or boundary judgment is wrong as false positive samples; the samples with no entity information in the prediction results of the entity extraction model and the test samples with no entity information as true negative samples; and the samples that do not contain entity information in the prediction results through the model output but should have entity information as false negative samples.

Select precision, recall, F value, and speed as the validation evaluation metrics for this experiment. Speed is an indicator proposed in this study to measure the running time of model training. Due to different datasets and hardware environments, the running time will be different. Therefore, in the following comparison experiments, the epoch of the total training should be controlled to be the same, and the speed value is the average training time (in seconds) required for each epoch:

$$Precision=\frac{T_{p}}{T_{p}+F_{p}},$$ (5-1)

$$Recall=\frac{T_{p}}{T_{p}+F_{n}},$$ (5-2)

$$F=\frac{2*Precision*Recall}{Precision+Recall}.$$ (5-3)

### 5.3 Analysis of results

The input for this experimental test is a chapter-based document sentence, and the output is the entity, the entity type, the start position of the entity, and the end position of the entity. The input and output samples are shown in Table 2.

**TABLE 2:** Test input and output sample table.

Input	Output		
	Entity	Type	Start and end location
**Cytotoxic** asterosaponins capable of promoting polymerization of tubulin from the starfish ***Culcita novaeguineae***	Cytotoxic	Bioactivity	0 and 8
	*Culcita*	Generic name	90 and 96
	*novaeguineae*	Specific name	98 and 109
Two new steroid glycosides from the far east starfish ***Hippasteria kurilensis***	*Hippasteria*	Generic name	54 and 64
	*kurilensis*	Specific name	66 and 75
Bromophycolides J-Q (1–8) were isolated from extracts of the Fijian **red alga *Callophycus serratus*** and identified with 1D and 2D NMR spectroscopy and mass spectral analyses	Red alga	Sample types	68 and 75
	*Callophycus serratus*	Generic name	77 and 96
As part of our search for bioactive substances from marine organisms systematically and assessing the chemical and biological diversities of seaweeds distributed along the Chinese coast, the **red alga** *Laurencia similis* was collected from **Sanya Bay, Hainan province**	Red alga	Sample types	184 and 191
	*Laurencia similis*	Generic name	193 and 209
	Sanya Bay, Hainan province	Sample source	230 and 255
**Polysiphonia urceolata** was collected **at the coast of Yantai, China**, in May 2008, and identified by Prof. Xiao Fan of the Institute of Oceanology, Chinese Academy of Sciences	*Polysiphonia urceolata*	Generic name	0 and 21
	At the coast of Yantai, China	Sample source	37 and 65

**FIGURE 4:**
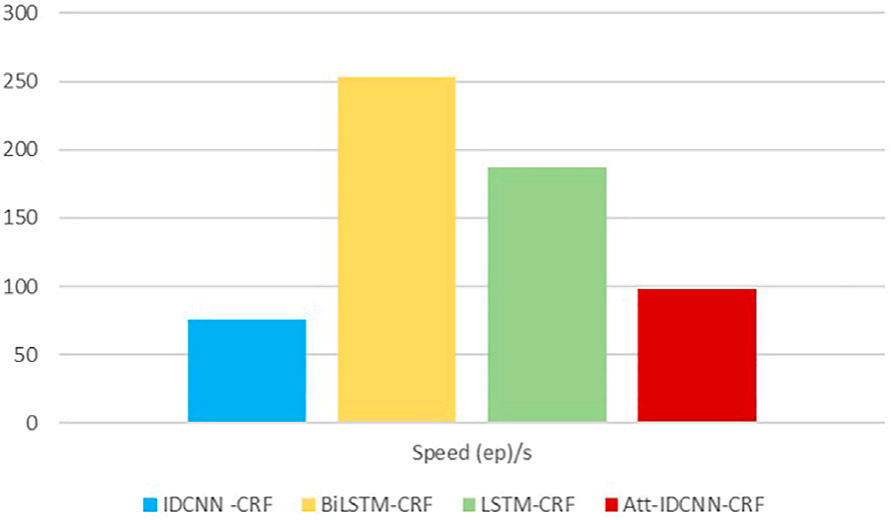
Chart of the speed metric.

The experimental results of this experiment are shown in Table 3. After analysis and comparison, the following conclusions can be drawn: compared with LSTM, BiLSTM-CRF, and other models applied to the named entity extraction, the improved attention-IDCNN-CRF on the original basis of the speed value of the model in the identification task of MNP source information is much higher than that of models LSTM-CRF and BiLSTM-CRF on average, as shown in Figure 4, and the recognition accuracy is significantly improved. Comprehensive experimental comparison results basically realized the main research objective of this study.

**TABLE 3:** Comparison of experimental results of recognition models.

Model	Precision	Recall	F	Speed (ep)/s
IDCNN-CRF	88.44	86.39	87.41	76
BiLSTM-CRF	90.57	89.35	89.96	253
LSTM-CRF	89.01	88.07	88.53	187
Att-IDCNN-CRF	92.18	90.71	91.44	98

**FIGURE 5:**
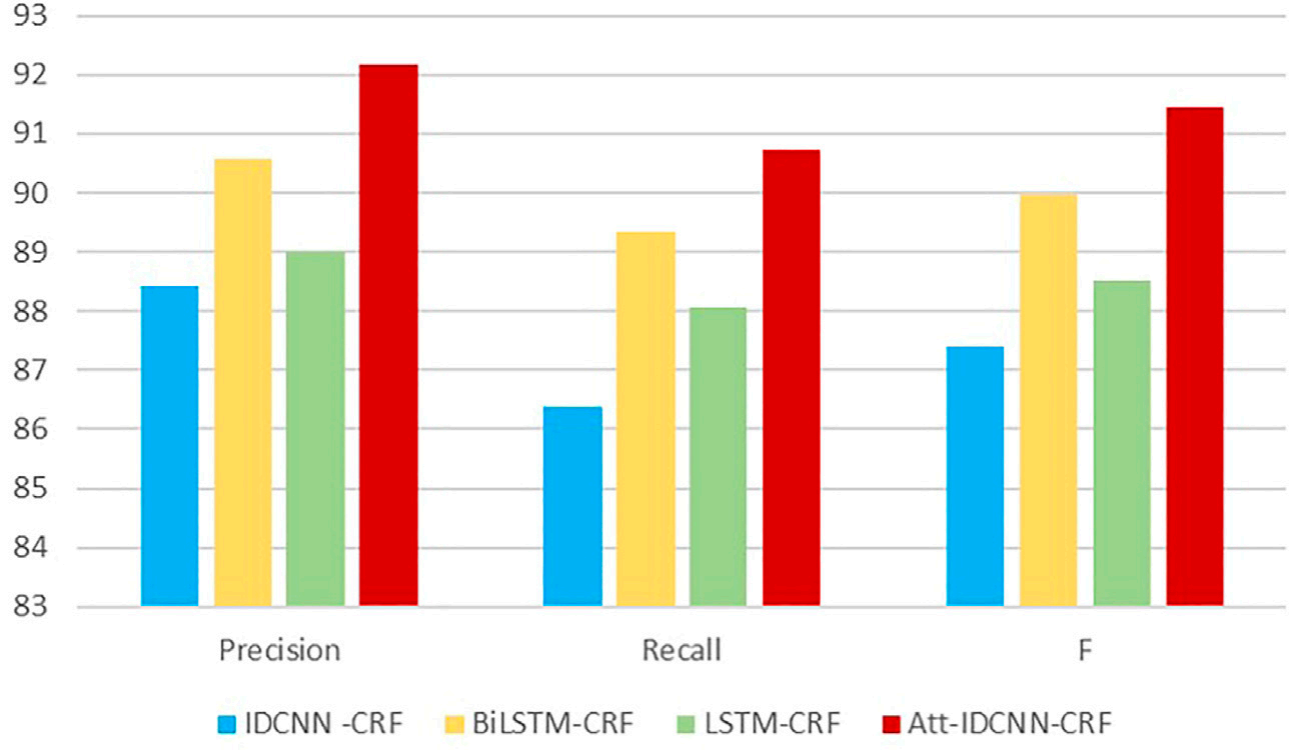
Chart of precision, recall, and F-statistic metrics.

Compared with the IDCNN-CRF model, the ATT-IDCNN-CRF model has a slight decrease in recognition speed. However, the attention mechanism can pay more attention to the weight of the chapter level than the traditional word/word level vector, and the F value is improved in the experiments of this study. Figure 5 shows that the IDCNN-CRF model with the attention mechanism has a better semantic expression ability.

## 6 Conclusion

This is a systematic study of named entity recognition methods for the MNP literature. First, it constructs a dataset of unstructured text in the field of MNPs. Second, an attention-based IDCNN-CRF named entity recognition model is improved and trained. By comparing multiple indicators, the advantages of the model are verified on the MNP dataset. The unique compound data information on MNPs is of great significance to related medical research, and it is very important to automatically extract relevant information. In the future, we will conduct more in-depth research in the field of named entity recognition of MNPs so that the training will be faster and the recognition effect will be more accurate.

## Data Availability

The raw data supporting the conclusion of this article will be made available by the authors, without undue reservation.
